# Are dialects socially learned in marmoset monkeys? Evidence from translocation experiments

**DOI:** 10.1371/journal.pone.0222486

**Published:** 2019-10-23

**Authors:** Yvonne Zürcher, Erik P. Willems, Judith M. Burkart

**Affiliations:** Department of Anthropology, University of Zurich, Winterthurerstrasse, Zürich, Switzerland; McGill University, CANADA

## Abstract

The acoustic properties of vocalizations in common marmosets differ between populations. These differences may be the result of social vocal learning, but they can also result from environmental or genetic differences between populations. We performed translocation experiments to separately quantify the influence of a change in the physical environment (experiment 1), and a change in the social environment (experiment 2) on the acoustic properties of calls from individual captive common marmosets. If population differences were due to genetic differences, we expected no change in the vocalizations of the translocated marmosets. If differences were due to environmental factors, we expected vocalizations to permanently change contingent with environmental changes. If social learning was involved, we expected that the vocalizations of animals translocated to a new population with a different dialect would become more similar to the new population. In experiment 1, we translocated marmosets to a different physical environment without changing the social composition of the groups or their neighbours. Immediately after the translocation to the new facility, one out of three call types showed a significant change in call structure, but 5–6 weeks later, the calls were no longer different from before the translocation. Thus, the novel physical environment did not induce long lasting changes in the vocalizations of the marmosets. In experiment 2, we translocated marmosets to a new population with a different dialect. Importantly, our previous work had shown that these two populations differed significantly in vocalization structure. The translocated marmosets were still housed in their original social group, but after translocation they were surrounded by the vocalizations from neighbouring groups of the new population. The vocal distance between the translocated individuals and the new population decreased for two out of three call types over 16 weeks. Thus, even without direct social contact or interaction, the vocalizations of the translocated animals converged towards the new population, indicating that common marmosets can modify their calls due to acoustic input from conspecifics alone, via crowd vocal learning. To our knowledge, this is the first study able to distinguish between different explanations for vocal dialects as well as to show crowd vocal learning in a primate species.

## Introduction

Population differences in vocalizations have been reported for many bird species [1], but also for mammals including primates [2]. Such population differences can be the result of environmental differences, if animals adapt to a vocal optimum in their local environment to increase signal transmission (i.e. environmental accommodation [3]). Alternatively, they can be the result of genetic differences, for instance when the latter lead to population level differences in vocal tract morphology. Finally, population differences in vocalizations can be the result of cultural transmission, i.e. vocal social learning or social accommodation. These latter cases are of particular interest because of their similarity with human dialects and potential implications for language evolution [2–4]. The terminology in vocal population differences is somewhat ambiguous in the animal literature. In humans, the word “dialect” is mainly used for lexical or grammatical differences, but according to Wolfram [5] it can include any language differences including pronunciation as well as language use. In birds, the term dialect was mainly used for vocal differences between neighbouring colonies that could interbreed, while differences between isolated populations were often termed “geographic variation”, although the two terms can be understood as two points on a continuum [6]. In primates, finally, dialect is often used for any acoustic differences between populations, including structural differences of the same calls (comparable to pronunciation differences in human dialects) (e.g. [7, 8, 9]). Here, we will follow the tradition of the primate literature and refer to population differences in acoustical call structure as dialects, following the definitions of [1, 5], but see [2] for a use of terminology more consistent with human literature.

Dialects in many birds are the result of cultural transmission, which is consistent with their propensity for vocal learning [1]. In contrast, in primates, vocal learning is rare [10] and current evidence that social factors play a role in population differences in vocal structure is indirect [2]. For instance, it has been argued that since vocal distance between chimpanzee populations is not correlated with geographical distance, genetic explanations are unlikely [11]. Likewise, available evidence supporting a role of vocal learning during ontogeny in callitrichid monkeys [12–18] may imply that population differences in these species are the result of vocal learning. However, whereas this evidence is certainly consistent with the idea that social accommodation is responsible for the emergence of population differences, we nevertheless cannot automatically assume that such ontogenetic effects are sufficient. Furthermore, environmental factors clearly can affect vocal structure in primates [3], including callitrichids [9, 19], and therefore are a likely source for population differences too [9, 20]. Population differences in vocal structure may thus well be the result of mechanisms other than social vocal learning in these species.

The gold standard for identifying the origin of population differences in behaviour, including vocalizations, is the use of translocation experiments [21]. These experiments allow to test whether population differences in vocalizations merely reflect genetic differences, as well as to separate the effect of environmental factors (by moving animals to a new location without changes in social constellation) and of social factors (by moving animals to a new population with a different dialect).

Our goal was to use translocation experiments to investigate the origin of population differences in common marmosets (*Callithrix jacchus*). Marmosets are callitrichid monkeys that show a high level of vocal plasticity [22, 23] and well-established population differences in vocalizations in the wild (pygmy marmosets (*Cebuella pygmaea*): [9]) and in captivity (common marmosets: [24]). We performed two translocation experiments with animals from two captive populations with known dialects [24] (experiment 1 and 2). If population differences were the result of genetic differences only, we predicted no change in vocalizations in response to any translocation. If they were the result of environmental accommodation, we expected their vocalizations to change permanently in a novel environment (experiment 1). If population differences were the result of social accommodation, we expected that animals translocated into a new population with a different dialect (experiment 2) would accommodate their vocalizations and become more similar to the vocalizations of the new population.

In experiment 1, we recorded three types of vocalizations of marmosets after translocation to a new facility: trill calls, phee calls and food calls. Trill calls are short distance contact calls, phee calls are long distance contact calls, and food calls are mainly produced in the context of discovering and sharing preferred food [25, 26]. We found that immediately after the translocation, food calls showed a significant change in call structure, but 5–6 weeks later, the calls were no longer different from before the translocation. Thus, the novel environment did not induce long lasting and permanent changes in the vocalizations. Consistent with these results, we also did not find any change in calls in an additional set of four individuals, 3–7 weeks after they had been translocated to a new physical environment (from their natal colony to a quarantine station without neighbours, see experiment 2).

In experiment 2, we translocated focal individuals from their original population via a quarantine station to a new target population. Importantly, the original and the target population show known vocal differences in trill calls, phee calls, and food calls, meaning that animals of each colony were acoustically more similar to other animals of their colony than to animals of other colonies [24]. We developed a statistical procedure to express and quantify vocal distances between the focal individuals and status- and sex-matched individuals from the target population *before* and *after* the translocation. We found that over a period of 16 weeks after translocation, the focal individuals were becoming more similar to the target population baseline with regard to trills and phee calls, and more dissimilar in food calls. Since in the new colony the focal individuals were still housed in the same social group as in the home colony and were exposed only passively to the vocalizations of the individuals from the target colony, this effect corresponds to crowd vocal learning recently reported in bats [27].

Together, the results of the translocation experiments revealed that marmoset calls are not strictly genetically fixed and that population differences are most likely the result of social vocal learning. Social vocal learning in common marmosets can thus occur by passive exposure to a different dialect, without direct social interaction, via crowd vocal learning.

## Results

### Experiment 1: Environmental accommodation

**Fig 1:**
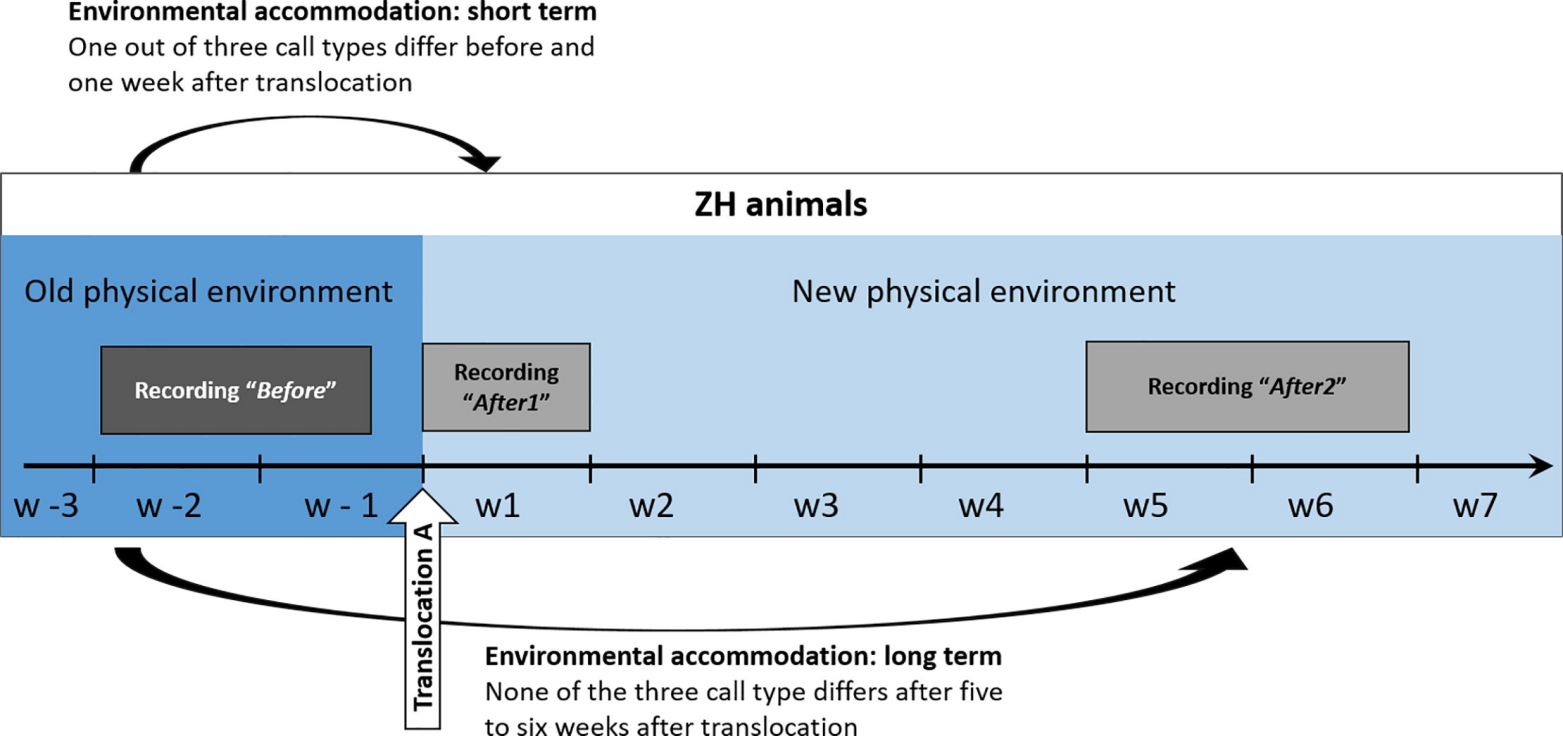
Timeline and comparison of calls before and after translocation A. The vocalizations of eight common marmosets were recorded in their familiar surrounding (old physical environment) over two weeks (“Before”). After this, the whole colony was moved to a new location. As soon as the eight monkeys were translocated to the new physical environment, they were recorded again during one week (“After1”), as well as after five to six weeks (“After2”). To investigate if changes in the physical environment lead to short term environmental accommodation, we compared the recordings “Before” to the recordings “After1” (upper black arrow). We found a significant change in food calls and a strong, but non-significant trend in phee calls, but no change in trill calls. To further analyse if these differences were stable over time (long-term environmental accommodation), we compared the recordings “Before” to the recordings “After2” (lower black arrow). None of the call types was different from before the translocation anymore, suggesting that the new environment did not lead to long-term accommodation.

To test whether common marmosets vocally accommodate to their environment, we recorded the vocalizations of eight common marmosets (referred to as “ZH animals”, a subset of the ZH colony) before and after they were translocated to a new building (a new acoustic environment, but the same social environment, “translocation A” in Fig 1). We recorded the animals regularly before and after the translocation event: immediately before and after the move (recordings “*Before”* and “*After1*” in Fig 1) and again 5–7 weeks later (“*After2*” in Fig 1).

We recorded three different call types (trill calls, phee calls, and food calls) and extracted 15 (phee—and food calls) or 17 (trill calls) parameters for each call, using the program Praat [28]. We did not include compound calls that show characteristics of two different call types, like trillphees. To reduce the number of parameters included in the analysis, we performed a principal component analysis (PCA) for each call type, resulting in 3–4 PC-Factors per call type, explaining 64.45%, 71.68% and 72.84% of the total variation respectively (see Table A in S1 File for factor loadings). First, we tested whether the calls given before and immediately after the translocation could be distinguished. We performed a crossed permutated Discriminant Function Analysis (pDFA) [29] and found that food calls differed significantly before vs. immediately after the translocation, but trill calls did not. Phee calls did not differ significantly, but showed a strong trend (Table 1).

**Table 1:** Short term environmental accommodation. In a sample of eight common marmosets from the ZH population, one of three call types showed a significant difference and one a strong trend to differ in the acoustic properties in the week immediately after translocation, when compared to the calls before the translocation. % of expected calls indicate the amount of correct classification if classification were random, % actual correct the amount of correct classification the pDFA reached with the data. Significant p-values are indicated in bold, trends in italics.

	Call type	Number of animals	Number of calls	% expected correct	% actual correct	p-value
**ZH animals** “Before”–“After1”	Trill call	8	311	56.28	59.59	0.325
	Phee call	8	607	54.09	60.30	*0*.*052*
	Food call	8	1292	56.23	68.51	**0.028**

To assess whether these changes were permanent, or perhaps only an artefact of increased stress levels due to the move, we recorded the same animals again 5–6 weeks after the translocation event (“*After2”)*. If the animals actually accommodated to the new physical environment, we predicted that the changes in calls should remain stable over time or become stronger, if accommodation takes more time than one week. We again performed a pDFA to test if the calls were still different from the calls before the move. We found that the changes observed immediately after the translocation did not persist over time, and calls from the period “*After2”* were no longer different from the period “*Before”* (Table 2). We thus did not find evidence for environmental accommodation after the translocation to the new facilities.

**Table 2:** Long-term environmental accommodation. ZH animals that were recorded again 5–6 weeks after translocation (“After2”), as well as four additional MA animals that were recorded 4–7 weeks after translocation (“NewPhys”), did not differ in their call structure compared to before the translocation. The new environment thus did not lead to persistent, long-term environmental accommodation. % of expected calls indicate the amount of correct classification if classification were random, % actual correct the amount of correct classification the pDFA achieved with the data.

	Call type	Number of animals	Number of calls	% expected correct	% actual correct	p-value
**ZH animals** “Before”—“After2”	Trill call	8	279	52.63	59.41	0.107
	Phee call	8	493	56.12	62.43	0.122
	Food call	8	895	52.77	54.62	0.402
**MA animals** “Before”–“NewPhys”	Trill call	4	278	64.29	73.31	0.389
	Phee call	4	833	57.05	64.05	0.156
	Food call	3	320	64.08	83.61	0.121

**Fig 2:**
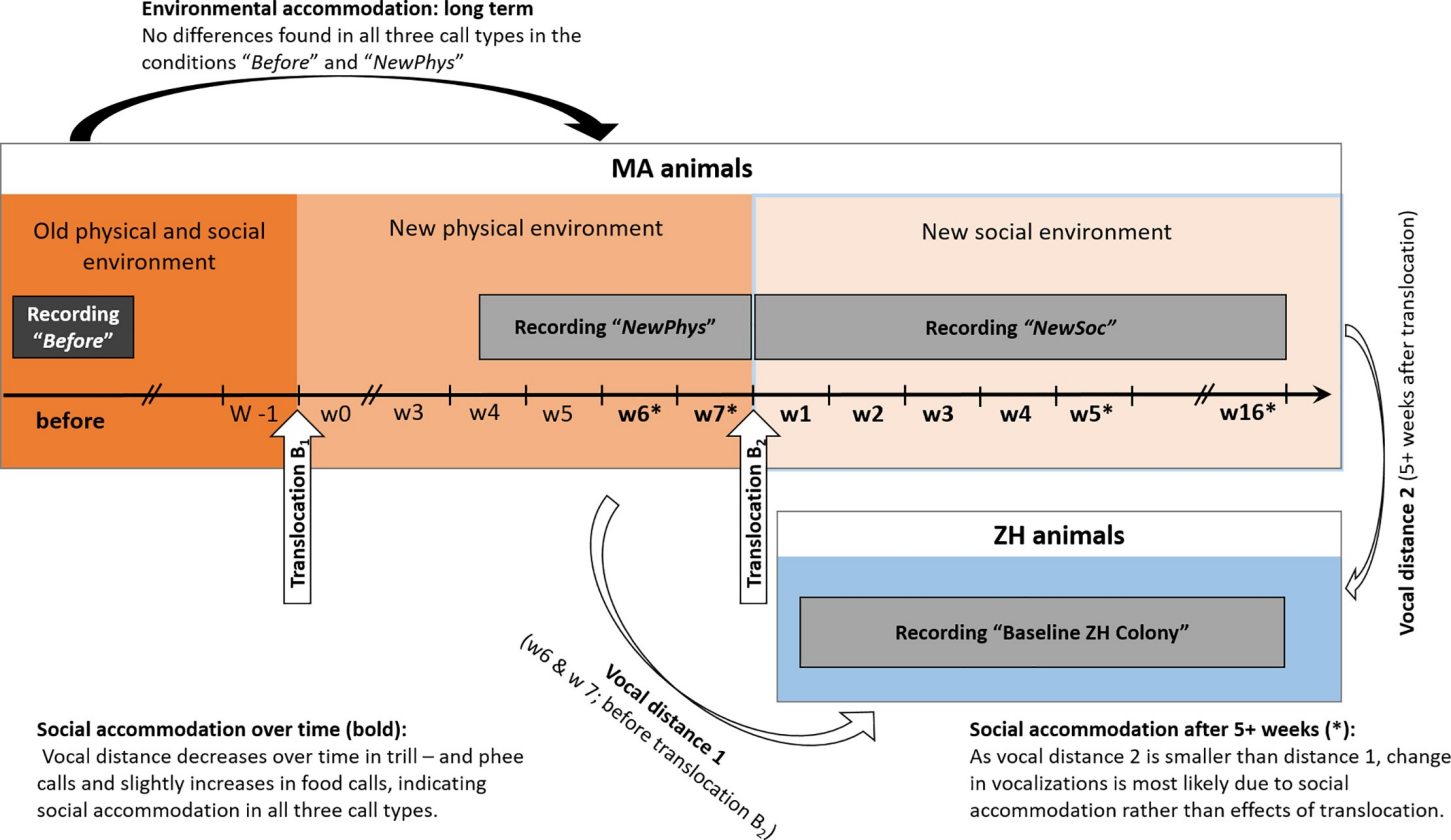
Timeline and comparisons of translocation B_1_ and B_2_. The vocalizations of the animals from MA were first recorded in their home colony (old physical and social environment, recording “Before”). After that, they were translocated to a new physical environment (“Translocation B_1_”, to a quarantine station, recording “NewPhys”), which allowed us to re-assess **long term environmental accommodation** (black arrow). Their calls did not differ before and after the translocation B_1_ to the new physical environment. Next, the animals were translocated to the ZH colony (“Translocation B_2_”, to a new social (and physical) environment, recording “NewSoc”). To quantify the **social accommodation over time**, we measured the vocal distance before any new social vocal contacts, during the weeks 6 & 7 after translocation B_1,_ as well as immediately after the animals arrived in the new colony and up to week 16, (weeks labelled in bold). To test for **social accommodation after 5+ weeks** of contact with the new colony (when potential short-term environmental accommodation effects must have disappeared), we compared the vocal distance (white arrows) between the animals from the ZH colony (“Baseline ZH Colony”) and the MA animals, immediately before Translocation B_2_ (vocal distance 1) and after they were 5+ weeks in the new social environment (after “Translocation B_2_”; vocal distance 2, weeks with *). We found that after translocation to the new social environment, the vocal distance between the MA and the ZH animals decreased in two call types and slightly increased in the third call type, as well as remained smaller / larger also after 5+ weeks, which is indicative of social accommodation.

We corroborated this pattern by also analyzing four additional individuals that were translocated from another colony in Madrid, Spain (referred to as “MA animals”) to a quarantine station, before they were integrated into the ZH colony (see below, Fig 2 and experiment 2). We recorded these animals both in their natal group (“*Before*”) and later on in the quarantine station (“*NewPhys*”). In the quarantine station, they were housed with a same-sex sibling from their natal group, thus in a new environment but without novel vocal input. We recorded the MA animals 4–7 weeks after arriving in the quarantine, i.e. after potential stress from the travel should have abated. We proceeded identically as with the calls from the ZH animals by first performing a PCA (resulting in 4 factors each, which in total explain 72.24%, 76.67% and 70.65% of the total variation respectively, see Table B in S1 File for factor loadings) and then a crossed pDFA to test whether the calls differed before and after the translocation. We did not find any structural differences in the calls of the MA animals for any of the three call types (Table 2), although the physical structures of the two recording facilities were substantially different (mainly a small wooden hut in Madrid vs a large concrete room in the quarantine). It therefore seems that translocating common marmosets to a physically different captive environment did not have any long lasting effects on their call structure.

### Experiment 2: Social accommodation

To test whether common marmosets showed social vocal accommodation after passive exposure to a new colony, i.e. crowd vocal learning, we translocated the four MA animals to the ZH colony (after they had spent seven weeks in quarantine, see Fig 2, translocation B_1_ and B_2_). In a previous study we could show that calls from animals of the MA population and the ZH population are significantly distinct [24]. It is important to note that this vocal differences between colonies indicates that animals from one colony are generally most similar to other animals of their colony but differ from animals of another colony. Animals of one colony share a dialect with one another, and by this also share typical acoustic features that make the colonies distinct.

After translocation B_2_, the translocated animals were still housed as sibling pairs, but in the same colony room as the ZH animals. This allowed for vocal and perhaps olfactory contact, but not for direct physical interaction or visual contact. We collected vocal recordings of the four MA animals as well as four status- and sex-matched ZH colony members (“*Baseline ZH colony*”). To quantify **vocal accommodation over time**, we measured vocal distance before each translocation as well as each week after translocation B_2_ for up to 16 weeks (see Fig 2).

First, we performed a PCA which resulted in 4 factors being retained, cumulatively accounting for 66.6.0%, 75.1%, and 70.8% of the total variation for trill calls, phee calls and food calls, respectively (see Table C in S1 File for factor loadings). Using these factors, we calculated the Euclidian distance between each measured call and its corresponding average from the four ZH animals. This resulted in several distance values per animal, per call type, and per week. To test how vocal distance developed over time in different call types, we performed a series of different linear models (see methods for details on model selection). The best model (Table 3) for trill calls revealed an interaction effect of sex and time, indicating that males showed stronger convergent accommodation than females. The best model for phee calls revealed a significant effect of both sex and time, whereas for food calls, it indicated a small but significant effect of time in the opposite direction (divergence) in food calls.

**Table 3:** Social accommodation over time. The effect of sex, exposure time and the interaction thereof on vocal distance (ln-transformed Euclidean distance) between the vocalisations of four translocated MA individuals and the call type specific average of the new population (“Baseline ZH colony”) for each call type. Parameter estimates, standard errors, and statistical significance are obtained from a linear mixed effects model. Significant (highest-order) effects are indicated with p-values in bold, trends in italics.

Trill				
	**B**	**SE**	**t**	**P**
**Intercept**	0.062	0.07		
**Sex**				
Female vs. Male	-0.137	0.088	-1.544	0.177
**Exposure time**	-0.052	0.008	-6.197	< 0.001
Sex * Exposure time				
Female vs Male interaction with week	0.032	0.009	3.494	**< 0.001**
N _obs._ = 515 from 4 individuals; R^2^_m_ = 0.114, R^2^_c_ = 0.146 χ^2^_MLT_ = 48.209, p< 0.05				
Phee				
	**B**	**SE**	**t**	**P**
**Intercept**	-0.008	0.062		
**Sex**				
Female vs. Male	-0.324	0.085	-3.801	0.023
**Exposure time**	-0.021	0.005	-3.932	**0.02**
N _obs._ = 1852 from 4 individuals; R^2^_m_ = 0.102, R^2^_c_ = 0.142, χ^2^_MLT_ = 10.2724, p< 0.05				
Food				
	**B**	**SE**	**t**	**P**
**Intercept**	-0.531	0.034		
**Sex**	0.100	0.046	2.160	0.151
Female vs. Male				
**Exposure time**	0.009	0.003	2.829	**0.005**
N _obs._ = 1759 from 4 individuals; R^2^_m_ = 0.009, R^2^_c_ = 0.0114, χ^2^_MLT_ = 9.846, p< 0.05				

**Fig 3:**
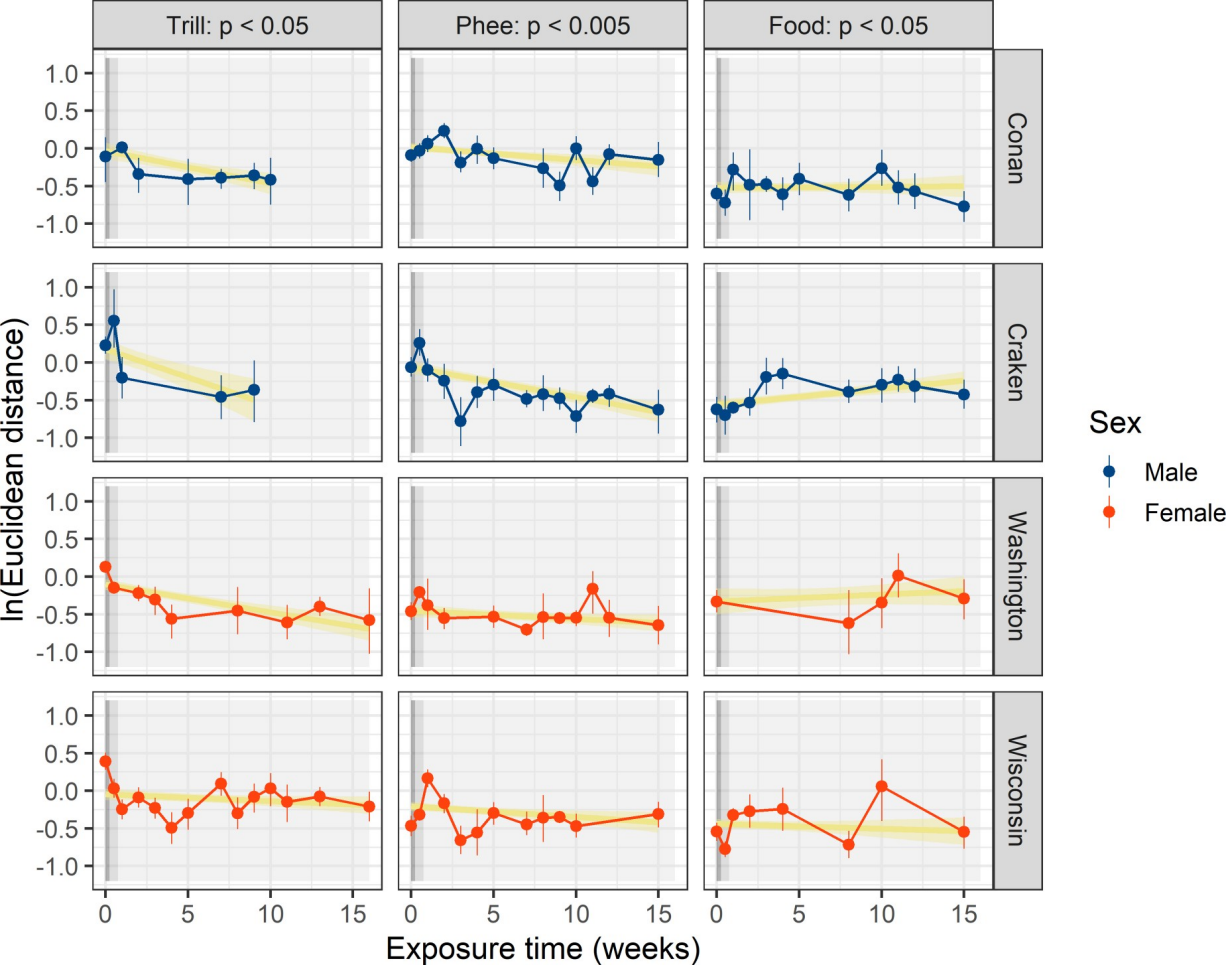
Social accommodation over time. Changes in vocal distance over time relative to the ZH population for the four translocated animals (rows), separately for each call type (columns). Dots represent weekly averaged values, calculated on a minimum of five recordings per individual per call type, with associated 95% (bootstrapped) confidence intervals represented by vertical error lines. Solid dark-yellow lines with shaded 95% confidence intervals represent the general pattern in change over time. Vocal distance decreases significantly over time in trill- and phee calls, and slightly increases in food calls.

Over all, the results show that for trill calls and phee calls, the vocal distance between the ZH and MA animals decreased over time, whereas it slightly increased in food calls. As the ZH baseline value was the same for all points in time in this analysis, the observed change in distance was most likely induced by the MA animals changing their vocalizations (see Table 3 and Fig 3).

Since the animals were at the same time also translocated to a novel environment (from quarantine to ZH colony room), some change in vocal structure may be due to short term environmental accommodation, as we had found in experiment 1. We therefore also analysed **social accommodation after 5+ weeks**, i.e. when potential short term environmental effects should have had disappeared.

**Fig 4:**
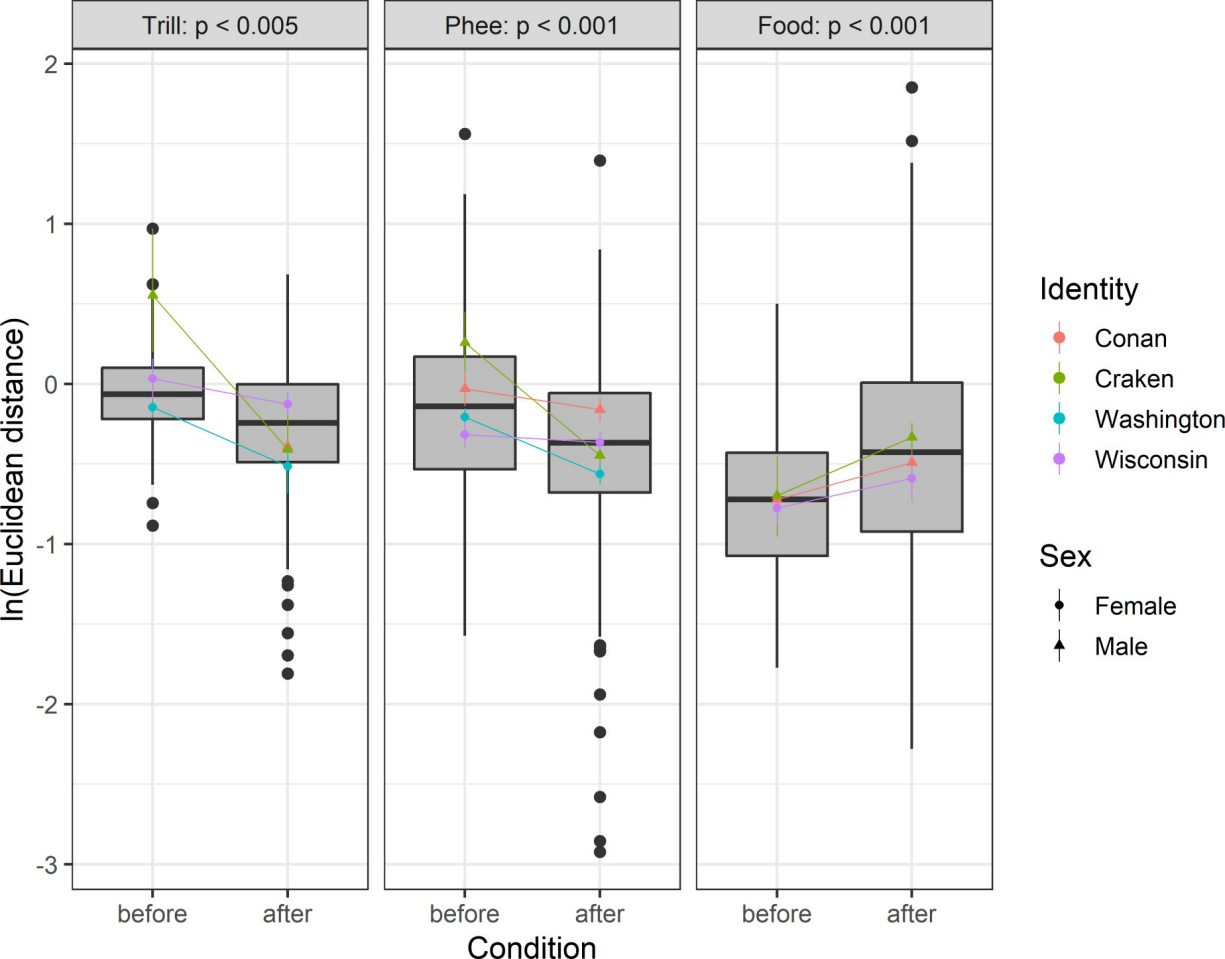
Social accommodation after 5+ weeks. The vocal distance was significantly larger immediately before translocation (last two weeks of the “*NewPhys*” condition = vocal distance 1 in Fig 2) than after 5+ weeks in the new colony (vocal distance 2 in Fig 2) for both phee- and trill calls. Food calls showed a slightly larger vocal distance after translocation, but effect size is small. Social accommodation thus persists over time and cannot be explained by short-term environmental accommodation.

We thus compared whether the vocal distance from the MA animals to the ZH baseline immediately before translocation (i.e. the last two weeks in the *“NewPhys”* condition, vocal distance 1 in Fig 2) was significantly larger than after 5+ weeks in the new colony (vocal distance 2 in Fig 2). We found that in both trill calls and phee calls, vocal distance was significantly smaller after the animals had spent at least 5 weeks in the new colony, whereas in food calls, the vocal distance increased slightly but significantly after 5+ weeks (see Fig 4 and Table D in S1 File for the models). Social accommodation effects thus persisted over time and were not an artefact of short-term environmental accommodation.

As we tested changes in vocal distance in call structure based on the combination of all call parameters, it is not straightforward to identify which parameter contributed most to the change in vocal distance. We nevertheless provide the average parameter for each individual and each call type before and after the translocation in the Table E–H in the S2 File for an overview, but emphasize that it is the combination of all rather than a single parameter that was responsible for the observed results.

## Discussion

Vocal dialects have been reported in marmosets in the wild [9] and in captivity [24], but to date it was not clear whether these population differences are the result of genetic differences, of environmental accommodation or of social vocal learning. Translocation experiments, as the ones reported here, provide an ultimate test to discriminate between these possibilities. In these experiments, individuals with their social partners are translocated to a different environment to test for environmental influences, or to a different population with a different dialect to test for social influences. The vocalizations of the individuals are constantly recorded, to allow a comparison of the call structures before and after translocation. To our knowledge, such studies are rare in the primate literature so far. Previous translocation studies found parallel changes, rather than divergence or convergence, in the vocalizations of pygmy marmosets after two social groups got in acoustic contact with each other [30]. Likewise, Rukstalis et al. could show that vocalizations of Wied’s black tufted-ear marmosets (*Callithrix kuhlii*) modified their phee calls after acoustic contact with unfamiliar individuals, although their analysis did not allow to quantify the direction or amount of change in the call properties [31]. The only study using a translocation approach in apes was done with chimpanzees, but unfortunately, crucial data of the subjects’ vocalizations before the translocation event was missing, and the study therefore had only limited potential to answer the question of vocal production learning in chimpanzees [32–34]. The current study is adding to this research by measuring the actual amount of accommodation, using a newly developed tool to quantify vocal distance. The results show that vocal dialects in common marmosets are not the result of genetic differences or environmental accommodation but arise due to social vocal learning.

Evidence for environmental accommodation after translocation was limited. We did find changes in call structure immediately after the translocation to a novel environment in one out of three call types, but these changes were not permanent and after five weeks, the calls were no longer different compared to before the translocation. This finding was corroborated in a second set of animals, where the change in environment was even larger (a small wooden hut or outdoor cage vs a large concrete room) yet no long-term effects could be detected. Thus, the environmental differences between the MA colony and the ZH colony could not account for the different dialects of these colonies. A likely explanation is that the changes documented during the first week after the translocation were due to the unfamiliar situation and potentially increased stress levels, rather than representing an adaptation to the acoustic properties of the new room. Nevertheless, environmental accommodation has been shown in callitrichid monkeys, including marmosets [19, 35]. Presumably, these environmental differences were more dramatic compared to the ones at stake in the present study, as environmental noise like wind and water (rivers, rainfall), as well as calls from other animals were much more prominent (and different) in the environments of wild populations [19] and mostly absent in our captive populations. It may thus well be that differences in acoustic properties in wild habitats can account for some amount of vocal differences, as has been shown for macaques and baboons [20, 36]. What our study suggests, however, is that such environmental factors are not necessary for the emergence of dialects in marmosets.

To test whether marmosets could socially learn a new vocal dialect, we translocated individuals between two colonies (MA and ZH) with different vocal dialects [24]. The vocal distance from the MA animals to the ZH baseline decreased over time after the MA animals had been translocated to the ZH colony, for two out of three call types. Social accommodation showed a slightly different pattern between males and females, and tended to be stronger in the former. However, as the sample size was rather small (two males and two females each), and the two same-sex animals were full siblings, we cannot conclude whether the observed difference is due to a general differences between the two sexes or whether it could be explained by family differences.

The second translocation included a change in social environment, but also in physical environment. Thus, short-term environmental accommodation may have played a role too. To exclude short-term environmental effects, we corroborated the results of social accommodation by comparing the calls immediately before translocation (*“NewPhys*”) with only the calls that were produced after 5 weeks or more in the new colony (when short-term environmental effects have disappeared, see experiment 1 on environmental accommodation). These additional analyses confirmed the presence of social accommodation: the vocal distance was again significantly smaller after 5+ weeks both in trill calls as well as in phee calls, and the change in vocal distance was again larger in males than in females (see Table D in the S1 File). In food calls, we found a small but significant increase in vocal distance. Food calls are mainly produced in the context where animals are willing to share food. As the MA subjects did not have any social bond to any of the ZH animals, and their only sharing partner was their sibling (with the same dialect), it could be that this divergence conserved the group identity and made it more distinct whom the caller was addressing.

In this study, we only investigated the vocal accommodation potential of the animals that were translocated (the MA animals). It may well be that the ZH individuals in acoustic contact with the new MA animals also changed their vocalizations in the direction of the MA animals. Unfortunately, we did not have enough recordings from the ZH animals to analyse this potential changes systematically as well. Note, however, that whether the ZH animals also changed their vocalizations or not is irrelevant for our conclusions, as the change of the MA animals towards the ZH animals per se shows that changes in vocalizations due to a social template is possible and occurs.

As marmoset vocalizations seem to be flexible to a certain degree and can change due to novel social inputs, we can also exclude genetic differences as a potential source of population differences, which suggests that social accommodation is thus the most likely explanation for the different dialects.

The effects found in this study were moderate. However, it is important to keep in mind that social accommodation in trill calls and phee calls occurred even though the MA animals were still housed with their siblings only, and were merely exposed to the vocalizations in the new colony. Learning a dialect by simply being exposed to it, without any direct interaction with animals of the new dialect, has so far, for nonhuman mammals, only been shown in bats [27]. Our results show that common marmosets too can learn a vocal dialect by simply being exposed to it, and thus show what Prat et al. call crowd vocal learning [27]. Impressively, common marmosets show this skill even as fully mature adults, and not in the developmental phase as it was the case in the bats. To fully understand the range of vocal accommodation in marmosets, it will be necessary to also study it when individuals are not only passively, but also actively exposed to the new dialect, i.e. when they are newly housed with animals with a different dialect and can have direct social contact with each other.

Overall, our results are in line with an increasing body of evidence suggesting some vocal learning potential in common marmosets. Both during ontogeny and as adults, marmosets modify their vocalizations due to social feedback. During ontogeny, exposure to calls of a caregiver plays an important role in the acquisition of vocalizations in immature marmosets [12]. Contrary to e.g. infant squirrel monkeys or macaques [37, 38], common marmosets with interrupted auditory feedback from caregivers did not make the full transition from a juvenile to an adult call repertoire, suggesting that vocal feedback is essential for these primates to produce correct vocalizations [14, 39]. Furthermore, similar to human infants, marmoset infants show an extensive babbling phase during their development [17, 18]. In pygmy marmosets, higher amounts of babbling leads to faster acquisition of adult-like calls [16], suggesting that babbling in marmosets might serve as practice for later vocal production just as in humans [40]. Intriguingly, immatures not only adjust their vocal development to parental feedback, but increasing evidence suggests that callitrichid parents themselves adjust their vocal feedback contingent on infant vocalizations [12, 41]. Adult marmosets show considerable flexibility in their call production too. They take part in turn taking exchanges, which requires precise coordination of their calls with their communication partner [42], and plan the timing of their calls to avoid predictable intervals of interfering background noise [43]. Finally, several species of the callitrichidae-family have been shown to modify their calls due to changes in their social environment, like new group members [30, 31] or new breeding partners [44]. Together, this body of evidence is fully consistent with our finding that genetic differences are not sufficient to account for differences in marmoset vocal structure, and that they even engage in crowd vocal learning. At the same time, it strongly suggests that immediate social context is a crucial factor for marmoset vocal learning. Accordingly, the effects of crowd vocal learning (convergence) were moderate, and only present in two out of three call types. Based on the recent literature, we hypothesise that social vocal learning effects will be much more prominent once the MA animals will be fully integrated in social groups with ZH animals, allowing for direct social contact.

Marmoset monkeys are increasingly used as a model species for neuroscientists to study the neuronal basis of behaviour, including vocal behaviour [45–47], in order to gain a better understanding of the evolutionary origin of human language. A detailed understanding of their vocalization behaviour, gained by careful observation and experimental manipulations, is a necessary precondition for this endeavour, and this study contributes to providing the necessary and solid base upon which neuroscientific studies can be built on [48]. Such studies continue to reveal that marmosets share a surprising amount of features with human language, and an intriguing hypothesis is that this may be linked to their social system. Like humans, marmosets are cooperative breeders, which might have shaped both their need as well as their social mind-set to favour vocal communication and coordination, in a similar way as it may have occurred during human evolution [49].

## Methods

### Subjects

We recorded at total of 10267 calls from 16 common marmosets for this study (4337 calls for environmental accommodation and 5930 calls for social accommodation; see Table 4). All animals were housed in pairs with either a sibling or a breeding partner in cages structured with a variety of wooden branches, ropes, tubes and other enriching material. The monkeys received food twice a day (vitamin enriched mush in the morning, mixed fruits and vegetables around midday) and various kind of animal proteins and insects and / or gum several times the day. Water was available ad libitum. The experiments were approved by the Kantonales Veterinärsamt Zürich, licence number ZH223/16.

**Table 4:** Overview of the number of calls used in the different analyses and of the individual contribution to the sample.

Translocation	ID	Origin	Sex	Trill calls	Phee calls	Food calls
A	Kaliper	ZH	M	87	67	178
A	Kapi	ZH	M	85	119	173
A	Kapo	ZH	M	51	121	105
A	Marlene	ZH	F	57	62	282
A	Tabor	ZH	M	22	166	348
A	Thilo	ZH	M	32	167	203
A	Vesta	ZH	F	59	26	117
A	Vito	ZH	M	39	80	183
	**Total**			**432**	**808**	**1589**
B_1_	Conan	MA	M	18	298	128
B_1_	Craken	MA	M	29	102	43
B_1_	Washington	MA	F	136	128	0
B_1_	Wisconsin	MA	F	95	305	149
	**Total**			**278**	**833**	**320**
B_2_	Conan	MA	M	114	557	501
B_2_	Craken	MA	M	45	315	545
B_2_	Washington	MA	F	113	298	149
B_2_	Wisconsin	MA	F	168	393	443
ZH Baseline	Gatto	ZH	M	16	304	555
ZH Baseline	Nautilus	ZH	M	33	164	76
ZH Baseline	Mibba	ZH	F	46	155	467
ZH Baseline	Lilly	ZH	F	27	86	360
**Total calls**				**562**	**2272**	**3096**

### Recording procedures and processing

For the vocal recordings, we used a Condenser Microphone CM16/CMPA and an AviSoft UltraSoundGate 116H together with the RECORDER software from AviSoft in all conditions. The microphone was directed toward the focal individual, which changed after 10 minutes. Recording sessions lasted 20 min, so every individual of a pair was the focal individual once per session. The order of focal animals was counterbalanced. The caller was identified visually by the observer and calls were labelled digitally during the recording using the labelling function provided by AviSoft RECORDER.

Each recording was visually scanned and each selected call saved as a separate sound file using AviSoft SASLab pro. Each single call was then measured in Praat [28], using a script by Reby and McComb ([50], adapted by E. Briefer), and each call was again visually controlled to ensure correct measuring. We measured 15 (phee calls and food calls) or 17 (trill calls) parameters for each call. These were for each call the fundamental frequency (F0) at the start and end of the call, mean, minimal and maximal F0, percentage of the call duration for which F0 was max, the absolute slope of F0, mean variation of F0 per second, the frequency values at the first, second and third quartiles of energy, the highest frequency of the whole spectrum, percentage of time this highest frequency is reached and jitter, as well as frequency modulation rate and frequency modulation extent for trill calls (for detailed description see [24, 51]).

For the analysis of experiment 1, we selected the first ten phee calls, five trills as well as twenty food calls from each recording of the ZH animals to reach a balanced sample. As the MA animals tended to vocalize only rarely in the “*NewPhys*” quarantine condition, we used every call they produced that passed our quality criteria for the subsequent analysis. Calls were excluded from the analysis if the identity of the caller could not be determined, if they overlapped with any other call or noise, if they were mixed with background noise or if we could not measure the whole call correctly in Praat. For the analysis of part 2, we only included animals in the data set if they contributed at least 5 calls per call type for a specific week.

### Study setup

#### Part 1: Environmental accommodation

Environmental accommodation was tested in 12 animals in total in two different settings. Eight marmosets (ZH animals, six males, two females) were recorded before and after the whole colony was moved to a new building (translocation A in Fig 1), whereas the other four marmosets (MA animals) were recorded after moving from their natal colony in Madrid, Spain to a quarantine station in Switzerland before being introduced to the new colony (translocation B_1_ in Fig 2).

The ZH animals were transferred from the old colony building (“*Before*” condition) to the new colony building (“*After1*” and “*After2*” condition). The new room was equipped with similar cages and enrichment as the old room, but differed in the surface materials of the walls and ceilings (metal and plastic vs wood) and in shape and size, supposedly resulting in rather different acoustic properties. To exclude social changes for the animals, the animals remained with their familiar partner in the new facilities and were housed in a similar social setting with their familiar neighbours.

All the animals were recorded repeatedly at three points in time: in the week before the moving process (“*Before*”), in the first week after the moving process (“*After1”*) and after five–six weeks (“*After2”*) (see Fig 1). In the “*Before*” condition, animals were shortly separated from their cage mate to elicit phee calls. In all other situations and for all other call types, animals were recorded in pairs. To elicit food calls, animals were presented with a mixture of highly preferred food such as meal worms, cashew nuts and gum in both conditions.

The MA animals were two males and two females, housed as a pair of brothers and a pair of sisters. They were initially living in their natal family group and were separated from their family either shortly before or at the time of the translocation B_1_. They were recorded at three points in time, over two weeks in their natal colony and family, seven months before translocation (“*Before*” condition), over 4 weeks in the quarantine (“*NewPhys*” condition, after Translocation B_1_) and over 16 weeks after introduction into the colony room of the new colony (“*NewSoc*”, after Translocation B_2_, see second part, social accommodation, and Fig 2). In the “*Before*” condition, the animals were recorded in pairs with another animal of their natal family group [see 24] and during the”*NewPhys”* and the “*NewSoc”* conditions with their sibling. To elicit food calls, animals were provided with different highly preferred food items like insects and pieces of banana. As the animals were rarely calling during the “*NewPhys*” situation, we provided a short play-back stimulus twice (females) and three times (males) to elicit calling behaviour. As playback stimuli we chose phee calls and food calls from former colony members in MA.

#### Part 2: Social accommodation

Social accommodation was tested in the four MA animals. After the time in the quarantine, the MA animals were introduced into the colony room housing the ZH population (Translocation B_2_). The animals were still housed with their respective sibling, and were visually and physically separated from the new population, but could vocally interact, therefore were exposed to a new vocal dialect [24]. The four MA animals were recorded over a period of 16 weeks in their home cage (“*NewSoc”)*, either alone to elicit phee calls or with their sibling. To elicit food calls, they were again provided with favourite food, like banana or insects.

The four MA animals were compared to a randomly chosen matched-control set of four ZH colony members. These animals, two males and two females, were unrelated, adult, non-breeding individuals that were all first housed with their family and later individually, as they were intended to become new breeding individuals (similar to the MA animals). Two of the ZH animals (one male and one female) were in acoustic contact with the MA animals, whereas the two other ZH animals were not, as they were housed in another colony room.

### Statistics

For all the analysis, we first reduced the dimensionality of our data. Therefore, we performed a principal component analysis on z-transformed values for each of the measured parameters. We extracted components with Eigenvalues greater than the 95% quantile value obtained from 10000 randomly generated datasets with equal sample size and dimensionality as our empirical data.

All the analysis were all performed with the statistics program “R”. pDFA were performed after a script by R. Mundry [29]. For Eigenvalue extraction of the PCAs we used the package “nFactors”[52], for calculating the LMEs the packages “lmerTest”[53] and “MuMIn” [54] and for creating the graphs “ggplot2” [55].

#### Part 1: Environmental accommodation

To test whether calls were different before and after translocation between environments in the ZH animals, we performed a permutated Discriminant Function analysis (pDFA) [29]. We used *condition (“Before” and “After1”)* as test factors and *individual* as control factor, and included the extracted PCA-factor as test variables. To test if changes were stable over time, we performed two crossed pDFAs, one for the ZH animals and one for the MA animals, with the condition “*Before*” and “*After*2” and “*Before*” and “*NewPhys*” as test factors and *individual* as control factors, including the extracted PCA-factor as test variables.

#### Part 2: Social accommodation

To assess whether the vocalisations of the four MA animals converged to their new population’s average vocalisation, we first performed a PCA. Next we weighted these component by the proportion of the total variance they explained, and calculated the Euclidean distance between each recorded vocalisation and its corresponding average vocalisation of the Zurich population. Mathematically we defined these three population average vocalisations as the centroids in the four-dimensional principal component space of each call type, and operationalised this as the average of the four Zurich individuals’ 10% trimmed-means along each of the component axes. We thus ended up with a single continuous value to express the extent to which each vocalisation of the MA animals differed from its call type specific average of the Zurich population, *i*.*e*. vocal distance.

To establish whether these vocal distances changed over time, we fitted a series of Linear Mixed Effects models (LME). Model parameters were approximated using maximum likelihood estimation, while model performance was assessed by likelihood-ratio tests against a null model consisting of the intercept and random effects only. In addition, the proportion of the total variance accounted for by each model was assessed by both marginal and conditional R^2^ values [56]. Following statistical convention, we only interpreted the highest order effect in which a predictor variable occurred whenever a significant interaction was present. To ensure parametric assumptions were met, we ln-transformed the outcome variable in all analyses.

Our model aimed to express vocal distance as a function of sex and week since introduction (where week 0 signifies recordings prior to auditory exposure to the local population (“*Before*”) and 0.5 signifies recordings in the “*NewPhys” condition after the first but before the second translocation*), and additionally considered second-order interactions between the two variables to investigate possible sex specific patterns.

To test whether calls were significantly different immediately before the second translocation and after 5+ weeks in the new colony, we performed a linear model expressing vocal distance as a function of condition (last two weeks before second translocation, *“NewPhys”* vs. 5+ weeks after translocation) and sex, as well as the interaction of the two. We choose 5+ weeks as a time range of comparison as we could show in the first part of the manuscript that potential changes in vocalizations due to the translocation should have been lost by then.

We used different statistical methods in experiment 1 and 2 due to the different questions we aimed to answer with the respective experiment. In experiment 1, we compared calls of the same individuals between different situations, and not to a specific baseline. We therefore applied the method commonly used for this type of comparison. For the experiment 2, we were not only interested in whether the calls of the MA animals change, but whether they became more similar to each other. We therefore developed a method to measure vocal distance between several individuals, to test for changes therein.

## Supporting information

S1 File This file contains the factor loadings for the different PCAs performed in the analysis (Table A—C), as well as the results of the models for social accommodation after week 5+ (Table D).
(DOCX)

S2 File The tables E—H provide the parameter average for each individual and each call type before and after the translocation. We would like to emphasize that it is the combination of all rather than a single parameter that was responsible for the observed results. Changes in any single parameter therefore only have a limited informative value.
(PDF)
