# Supplementary material for: Are dialects socially learned in marmoset monkeys? Evidence from translocation experiments
Source: PLoS One. 2019 Oct 23;14(10):e0222486. doi: 10.1371/journal.pone.0222486 (PMC6808547; doi:10.1371/journal.pone.0222486)
Supplement: S1 File — This file contains the factor loadings for the different PCAs performed in the analysis (Table A—C), as well as the results of the models for social accommodation after week 5+ (Table D). (DOCX) [file pone.0222486.s001.docx]

# Supplementary material 1

Experiment 1, Environmental accommodation

**Table A**: Factor loadings of the call parameters on the extracted PCA factors (PC 1- PC 4) for the ZH animals in the analysis of the Experiment 1: Environmental accommodation.

|  | **Trill calls** | | | **Phee calls** | | | | **Food calls** | | | |
| --- | --- | --- | --- | --- | --- | --- | --- | --- | --- | --- | --- |
| Parameter | PC1 | PC2 | PC3 | PC1 | PC2 | PC3 | PC4 | PC1 | PC2 | PC3 | PC4 |
| sound duration | -0.26 | -0.15 | -0.10 | -0.07 | 0.46 | -0.13 | 0.08 | 0.04 | 0.52 | -0.07 | 0.03 |
| Mean F0 | -0.39 | 0.19 | 0.07 | -0.43 | -0.02 | 0.08 | -0.01 | 0.43 | 0.00 | 0.12 | 0.04 |
| F0 start | -0.25 | 0.41 | 0.13 | -0.37 | 0.07 | 0.15 | 0.31 | 0.42 | -0.01 | 0.11 | -0.11 |
| F0 end | -0.41 | 0.08 | 0.05 | -0.37 | 0.00 | -0.06 | -0.34 | 0.39 | 0.08 | 0.13 | 0.24 |
| Max F0 | -0.42 | 0.07 | 0.04 | -0.41 | -0.04 | 0.02 | -0.15 | 0.42 | -0.01 | 0.11 | -0.10 |
| percTime Max F0 | -0.17 | -0.15 | 0.10 | -0.06 | 0.22 | -0.34 | -0.66 | -0.04 | -0.44 | 0.02 | 0.47 |
| Min F0 | -0.25 | 0.41 | 0.14 | -0.39 | 0.11 | 0.12 | 0.18 | 0.39 | 0.08 | 0.13 | 0.24 |
| F0 abs slope | -0.21 | -0.35 | -0.04 | 0.00 | -0.53 | 0.11 | -0.04 | 0.13 | -0.44 | 0.06 | -0.32 |
| F0 Var | -0.25 | -0.37 | -0.07 | -0.02 | -0.52 | 0.11 | -0.07 | 0.07 | 0.02 | -0.02 | -0.66 |
| Q25perc | -0.06 | -0.08 | 0.57 | -0.40 | -0.05 | -0.07 | 0.18 | 0.31 | -0.07 | -0.34 | 0.03 |
| Q50perc | 0.14 | -0.21 | 0.53 | -0.16 | -0.24 | -0.54 | 0.11 | 0.15 | -0.15 | -0.62 | 0.00 |
| Q75perc | 0.04 | -0.20 | 0.51 | 0.04 | -0.25 | -0.58 | 0.16 | 0.08 | -0.07 | -0.61 | 0.01 |
| Fpeak | -0.06 | -0.05 | -0.01 | 0.00 | 0.12 | -0.25 | 0.06 | 0.02 | 0.15 | -0.01 | 0.04 |
| percTime of max intensity | -0.15 | -0.02 | -0.01 | 0.01 | 0.09 | -0.32 | 0.39 | -0.05 | -0.34 | 0.08 | 0.22 |
| jitter | 0.21 | 0.02 | -0.14 | -0.14 | -0.16 | -0.06 | -0.26 | 0.02 | -0.41 | 0.20 | -0.22 |
| Fm Rate | 0.15 | 0.29 | 0.17 |  |  |  |  |  |  |  |  |
| FM extend | -0.24 | -0.37 | -0.13 |  |  |  |  |  |  |  |  |
|  |  |  |  |  |  |  |  |  |  |  |  |
| Eigenvalue | 5.46 | 3.35 | 2.15 | 5.00 | 3.14 | 1.46 | 1.15 | 5.25 | 2.16 | 1.83 | 1.68 |
| % of total variance | 32.12 | 19.70 | 12.63 | 33.36 | 20.90 | 9.72 | 7.70 | 35.03 | 14.41 | 12.18 | 11.22 |

|  | **Trill calls** | | | | **Phee calls** | | | | **Food calls** | | | |
| --- | --- | --- | --- | --- | --- | --- | --- | --- | --- | --- | --- | --- |
| Parameter | PC1 | PC2 | PC3 | PC4 | PC1 | PC2 | PC3 | PC4 | PC1 | PC2 | PC3 | PC4 |
| sound duration | 0.08 | -0.15 | -0.32 | -0.49 | 0.12 | -0.45 | 0.07 | -0.10 | -0.06 | -0.36 | -0.07 | 0.22 |
| Mean F0 | -0.40 | -0.06 | 0.01 | -0.07 | 0.42 | -0.06 | 0.10 | 0.08 | -0.42 | 0.04 | 0.03 | 0.10 |
| F0 start | -0.38 | -0.04 | 0.12 | -0.02 | 0.32 | 0.27 | -0.20 | 0.25 | -0.40 | 0.10 | -0.11 | 0.12 |
| F0 end | -0.37 | -0.14 | -0.03 | -0.05 | 0.38 | -0.18 | 0.19 | -0.04 | -0.40 | -0.04 | 0.21 | 0.08 |
| Max F0 | -0.39 | 0.01 | -0.01 | -0.14 | 0.38 | -0.16 | 0.22 | 0.05 | -0.40 | 0.08 | -0.13 | 0.15 |
| percTime Max F0 | 0.02 | -0.19 | -0.12 | 0.17 | 0.03 | -0.26 | 0.22 | -0.49 | 0.02 | -0.44 | 0.02 | 0.29 |
| Min F0 | -0.38 | -0.15 | 0.09 | 0.05 | 0.35 | 0.22 | -0.21 | 0.15 | -0.40 | -0.02 | 0.22 | 0.06 |
| F0 abs slope | -0.08 | 0.53 | -0.08 | 0.12 | -0.07 | 0.31 | 0.57 | 0.03 | 0.01 | 0.06 | -0.64 | 0.28 |
| F0 Var | -0.09 | 0.52 | 0.04 | 0.01 | -0.04 | 0.24 | 0.63 | 0.06 | 0.02 | 0.49 | -0.42 | -0.22 |
| Q25perc | -0.39 | -0.06 | 0.07 | 0.01 | 0.41 | 0.01 | -0.01 | 0.06 | -0.33 | -0.05 | -0.05 | -0.35 |
| Q50perc | -0.26 | -0.02 | -0.38 | 0.19 | 0.33 | 0.15 | 0.04 | -0.29 | -0.26 | -0.27 | -0.30 | -0.41 |
| Q75perc | -0.04 | -0.10 | -0.54 | 0.29 | 0.10 | 0.33 | -0.19 | -0.51 | 0.03 | -0.47 | -0.15 | -0.53 |
| Fpeak | 0.02 | -0.09 | -0.20 | -0.41 | 0.04 | -0.26 | -0.02 | -0.15 | -0.05 | 0.02 | -0.09 | -0.08 |
| percTime of max intensity | -0.05 | 0.20 | 0.06 | 0.39 | 0.00 | -0.05 | -0.06 | -0.42 | -0.11 | 0.21 | -0.05 | -0.06 |
| jitter | 0.04 | 0.01 | -0.53 | 0.26 | 0.04 | 0.43 | -0.03 | -0.32 | 0.00 | -0.28 | -0.40 | 0.33 |
| Fm Rate | 0.03 | -0.19 | 0.26 | 0.34 |  |  |  |  |  |  |  |  |
| FM extend | -0.11 | 0.49 | -0.14 | -0.26 |  |  |  |  |  |  |  |  |
|  |  |  |  |  |  |  |  |  |  |  |  |  |
| Eigenvalue | 6.00 | 2.60 | 2.07 | 1.60 | 5.28 | 3.21 | 1.80 | 1.21 | 5.59 | 2.07 | 1.69 | 1.24 |
| % of total variance | 35.32 | 15.30 | 12.19 | 9.44 | 35.19 | 21.42 | 11.99 | 8.06 | 37.30 | 13.82 | 11.24 | 8.29 |

**Table B**: Factor loadings of the call parameters on the extracted PCA factors (PC 1- PC 4) for the MA animals in the analysis of the Experiment 1: Environmental accommodation.

Experiment 2, Social accommodation

**Table C**: Factor loadings of the call parameters on the extracted PCA factors (PC 1- PC 4) for the MA and ZH baseline animals used in the analysis of the Experiment 2: Social accommodation.

|  | **Trill calls** | | | | **Phee calls** | | | | **Food calls** | | | |
| --- | --- | --- | --- | --- | --- | --- | --- | --- | --- | --- | --- | --- |
|  | PC1 | PC2 | PC3 | PC4 | PC1 | PC2 | PC3 | PC4 | PC1 | PC2 | PC3 | PC4 |
| sound duration | 0.09 | -0.27 | -0.40 | 0.30 | 0.04 | -0.53 | 0.11 | -0.01 | 0.07 | -0.08 | -0.39 | 0.33 |
| Mean F0 | -0.43 | -0.16 | 0.00 | 0.03 | 0.40 | -0.05 | 0.11 | -0.15 | 0.41 | 0.09 | 0.07 | 0.03 |
| F0 start | -0.43 | 0.09 | 0.14 | -0.01 | 0.35 | 0.16 | -0.24 | -0.18 | 0.41 | -0.06 | 0.13 | 0.06 |
| F0 end | -0.37 | -0.26 | -0.15 | 0.09 | 0.36 | -0.16 | 0.17 | -0.07 | 0.36 | 0.33 | 0.00 | 0.02 |
| Max F0 | -0.38 | -0.29 | -0.03 | 0.04 | 0.36 | -0.15 | 0.27 | -0.13 | 0.41 | -0.05 | 0.12 | 0.04 |
| percTime Max F0 | 0.10 | -0.21 | -0.35 | 0.11 | 0.02 | -0.33 | 0.20 | 0.27 | -0.03 | 0.25 | -0.19 | -0.59 |
| Min F0 | -0.44 | 0.10 | 0.07 | 0.02 | 0.36 | 0.13 | -0.25 | -0.15 | 0.36 | 0.32 | 0.00 | 0.03 |
| F0 abs slope | 0.09 | -0.28 | 0.45 | -0.29 | -0.05 | 0.42 | 0.50 | -0.04 | 0.14 | -0.44 | 0.31 | -0.21 |
| F0 Var | 0.10 | -0.37 | 0.31 | -0.28 | -0.04 | 0.37 | 0.55 | -0.07 | 0.07 | -0.46 | 0.34 | 0.19 |
| Q25perc | -0.21 | -0.12 | 0.31 | 0.30 | 0.39 | -0.02 | 0.06 | 0.00 | 0.37 | -0.09 | -0.17 | -0.06 |
| Q50perc | 0.18 | -0.04 | 0.37 | 0.45 | 0.35 | 0.09 | 0.10 | 0.27 | 0.25 | -0.34 | -0.34 | -0.27 |
| Q75perc | 0.11 | 0.06 | 0.28 | 0.54 | 0.15 | 0.23 | -0.14 | 0.59 | 0.04 | -0.36 | -0.49 | -0.27 |
| Fpeak | 0.04 | -0.06 | -0.10 | 0.36 | 0.02 | -0.19 | 0.09 | 0.23 | 0.03 | -0.10 | -0.11 | 0.00 |
| percTime of max intensity | 0.04 | -0.19 | 0.02 | 0.02 | 0.04 | -0.06 | 0.12 | 0.57 | 0.02 | 0.17 | 0.26 | -0.44 |
| jitter | -0.06 | 0.24 | 0.20 | 0.09 | 0.12 | 0.32 | -0.31 | 0.16 | -0.04 | -0.12 | 0.32 | -0.35 |
| Fm Rate | -0.07 | 0.36 | 0.00 | -0.06 |  |  |  |  |  |  |  |  |
| FM extend | 0.11 | -0.47 | 0.05 | -0.01 |  |  |  |  |  |  |  |  |
|  |  |  |  |  |  |  |  |  |  |  |  |  |
| Eigenvalue | 4.70 | 3.2 | 1.96 | 1.45 | 5.58 | 2.61 | 1.92 | 1.16 | 5.61 | 2.05 | 1.57 | 1.3 |
| % of total variance | 27.7 | 18.9 | 11.5 | 8.5 | 37.2 | 17.4 | 12.8 | 7.8 | 37.4 | 13.7 | 10.5 | 9.3 |

**Table D: Social accommodation after 5+ weeks:** The effect of condition (shortly before translocation or 5+ weeks after translocation), sex, and the respective interactions on vocal distance (ln-transformed Euclidean distance) between the vocalisations of four translocated individuals and the call type specific average of the new population. Parameter estimates, standard errors, and statistical significance are obtained from a linear mixed effects model. Significant (highest-order) effects are indicated with p-values in bold.

| Trill | |  |  |  |
| --- | --- | --- | --- | --- |
|  | **B** | **SE** | **t** | **P** |
| **Intercept** | 0.5604 | 0.2536 |  |  |
| **Condition** | -0.9590 | 0.2459 | -3.9 | < 0.001 |
| **Sex** | -0.6063 | 0.2801 | -2.164 | 0.046 |
| **Condition * Sex** |  |  |  |  |
| Before and after translocation by sex | 0.7083 | 0.2530 | 2.8 | **0.006** |
| N _obs._= 515 from 4 individuals; R^2^_m_= 0.128, R^2^_c_= 0.251 χ^2^_MLT_= 8.095, p< 0.05 | | | | |
|  |  |  |  |  |
| Phee | |  |  |  |
|  | **B** | **SE** | **t** | **P** |
| **Intercept** | 0.05282 | 0.09502 |  |  |
| **Condition** | -0.34725 | 0.05295 | -6.558 | < 0.001 |
| **Sex** | -0.44615 | 0.13333 | -3.346 | 0.048 |
| **Condition * Sex** |  |  |  |  |
| Before and after translocation by sex | 0.27506 | 0.07434 | 3.700 | **< 0.001** |
| N _obs._= 1001 from 4 individuals; R^2^_m_= 0.085, R^2^_c_= 0.135, χ^2^_MLT_= 12.7693 , p< 0.05 | | | | |
|  |  |  |  |  |
| Food | |  |  |  |
|  | **B** | **SE** | **t** | **P** |
| **Intercept** | -0.709 | 0.084 |  |  |
| **Condition** | 0.247 | 0.069 | 3.561 | **< 0.001** |
| N _obs._= 591 from 3 individuals; R^2^_m_= 0.0244, R^2^_c_= 0.0504, χ^2^_MLT_= 9.846, p< 0.05 | | | | |
