# Supplementary material for: Are dialects socially learned in marmoset monkeys? Evidence from translocation experiments
Source: PLoS One. 2019 Oct 23;14(10):e0222486. doi: 10.1371/journal.pone.0222486 (PMC6808547; doi:10.1371/journal.pone.0222486)
Supplement: S2 File — The tables E—H provide the parameter average for each individual and each call type before and after the translocation. We would like to emphasize that it is the combination of all rather than a single parameter that was responsible for the observed results. Changes in any single parameter therefore only have a limited informative value. (PDF) [file pone.0222486.s002.pdf]

Supplementary Material 2

We tested changes in vocal distance in call structure based on the combination of all call parameters, therefore it is not straightforward to identify which parameter contributed most to the change in vocal distance. We nevertheless provide the average parameter for each individual and each call type before and after the translocation in the supplementary material 2 (Table S2.1 – S2-4) for an overview, but would like to emphasize that it is the combination of all rather than a single parameter that was responsible for the observed results. Changes in any single parameter therefore only have a limited informative value.

Table E Average value of each call parameter from the ZH animal in experiment 1 from *"Before"* and *"After1"*

		Kapi		Kapo		Tabor		Thilo		Kaliper		Marlene		Vesta		Vito	
		Before	*After1*	*Before*	*After1*	*Before*	*After1*	*Before*	*After1*	*Before*	*After1*	*Before*	*After1*	*Before*	*After1*	*Before*	*After1*
Trill calls	sound duration	0.472	0.522	0.446	0.599	0.583	0.565	0.498	0.508	0.517	0.579	0.645	0.735	0.415	0.383	0.776	0.451
	Mean F0	6733.6	5516.2	6036.9	6068.1	6376.5	6582.8	6631.9	6634.3	6826.3	6450.0	6561.4	6353.6	5979.2	5909.6	6923.8	6055.9
	F0 start	6198.9	4890.4	5610.8	5357.1	5355.1	5724.6	6019.5	6342.7	6536.9	5916.2	5850.3	5432.8	5632.0	5679.6	5168.3	5507.8
	F0 end	7025.2	5718.4	6086.5	6333.3	6961.5	6925.2	7057.1	6916.0	6877.7	6602.8	6982.8	6784.7	6249.9	6079.5	7712.5	6251.2
	Max F0	7120.7	5908.6	6255.0	6417.8	7101.7	7074.8	7122.8	6939.7	7077.3	6826.6	7037.2	6902.8	6303.6	6186.3	7839.0	6321.8
	% Time Max F0	77.1	72.9	66.7	76.3	73.9	82.1	81.4	66.1	67.4	69.3	77.7	80.3	68.2	60.1	83.0	71.3
	Min F0	6180.1	4869.7	5592.0	5357.1	5292.8	5724.6	6015.0	6231.9	6493.0	5833.1	5842.4	5406.9	5597.5	5613.6	5168.3	5503.7
	F0 abs slope	2958.0	3073.0	2385.0	2654.5	3957.0	3424.8	3105.2	2392.2	2251.0	2864.5	2470.2	2761.0	2165.7	2301.6	4308.5	2762.8
	F0 Var	1883.1	2150.6	1405.3	1874.3	2821.7	2356.0	2126.8	1431.2	1417.1	1921.9	1656.0	2049.2	1242.9	1318.0	3348.4	1688.7
	Q25%	8017.7	7586.3	6446.2	6746.6	6876.4	7959.0	6488.2	6658.3	7093.1	6747.6	6702.5	7637.5	6290.2	8454.0	7035.5	6623.3
	Q50%	10822.4	11807.5	9977.1	10265.0	9265.7	9630.5	7861.4	8216.6	8756.7	8888.5	9293.8	9123.6	9563.2	11241.1	8137.6	9696.6
	Q75%	15473.7	16793.8	12508.1	13324.6	12657.5	12845.7	11361.9	13216.4	12689.6	13287.1	12841.3	12085.5	12978.0	14749.5	11919.1	12492.1
	Fpeak	200.6	229.9	214.2	204.7	219.5	222.4	174.0	158.4	229.7	215.7	203.1	211.1	203.2	198.8	338.2	217.5
	% Time of max intensity	22.8	28.8	28.9	53.0	35.2	19.5	26.1	28.9	29.7	32.1	33.5	49.8	28.0	25.2	65.9	26.9
	jitter	0.019	0.021	0.023	0.027	0.019	0.023	0.023	0.016	0.021	0.018	0.024	0.020	0.023	0.023	0.015	0.020
	Fm Rate	2.2	1.7	2.0	2.0	1.5	2.6	2.1	2.2	2.9	2.0	1.8	1.2	2.5	2.7	0.8	2.0
	FM extend	1355.9	1664.3	1117.8	1223.8	2822.0	1405.4	1432.3	727.4	744.3	1271.9	1468.3	2287.1	949.6	601.9	4724.1	994.0

Phee Calls	sound duration	1.089	0.971	0.962	1.165	0.787	0.878	1.231	1.014	1.305	1.358	1.097	1.324	1.963	1.318	1.137	1.672
	Mean F0	7244.2	6804.8	7747.3	7359.7	6979.1	6977.2	7507.5	7560.4	7856.1	7455.8	7690.0	7546.2	7095.3	7214.6	7957.3	7765.0
	F0 start	6518.2	5866.6	6833.5	6276.5	6238.7	5983.4	6623.9	6553.1	7130.3	6634.4	6855.1	6736.4	6511.8	6234.8	7310.6	6765.2
	F0 end	7627.5	7338.1	8119.8	7806.6	6899.9	7003.9	7592.4	7824.8	8168.0	7742.8	8033.1	7701.9	6719.9	7319.5	8165.3	7862.4
	Max F0	7858.1	7477.6	8393.8	8058.1	7450.3	7438.7	8116.8	8118.4	8438.6	8061.4	8272.5	8127.7	7430.9	7663.0	8479.3	8116.0
	% Time Max F0	84.0	85.2	79.4	82.9	71.6	77.3	79.4	79.8	86.6	84.7	77.2	78.3	86.6	83.8	80.5	71.3
	Min F0	6478.8	5859.4	6754.1	6251.2	6190.6	5979.6	6545.7	6549.7	7127.5	6629.2	6791.7	6731.9	6359.1	6229.4	7115.0	6735.8
	F0 abs slope	2146.6	2725.5	2892.4	2719.9	3022.1	2895.4	2200.6	2524.8	1454.3	1505.2	2293.4	1758.2	982.2	1767.2	1827.9	1381.9
	F0 Var	1691.5	2171.6	2209.9	2123.2	2202.5	2167.5	1734.4	1889.0	1169.3	1212.6	1844.2	1347.1	687.2	1391.6	1483.2	1091.0
	Q25%	7195.4	6816.4	7700.6	7351.8	6976.3	7004.5	7314.8	7408.4	7690.7	7370.8	7479.8	7553.1	7126.9	7287.9	7729.7	7753.8
	Q50%	8151.8	8358.6	8596.7	8472.0	7557.0	7705.0	7713.7	7794.9	7995.7	7940.8	7856.9	8679.3	7333.5	8097.6	8192.1	7943.9
	Q75%	13085.6	13276.4	12133.5	12678.7	10402.2	11439.4	9525.2	9399.7	9097.2	10925.2	8482.6	13598.8	10371.8	12785.2	10460.8	9029.7
	Fpeak	234.9	277.5	233.9	263.9	212.0	231.6	255.2	252.8	257.1	346.4	229.7	286.0	276.7	289.1	253.4	245.3
	% Time of max intensity	46.8	48.0	48.5	47.9	48.0	48.3	39.5	41.1	46.4	46.6	47.6	50.1	52.1	58.8	32.3	53.9
	jitter	0.007	0.005	0.008	0.007	0.006	0.005	0.006	0.007	0.006	0.006	0.006	0.005	0.005	0.005	0.012	0.007
Food Call	sound duration	0.099	0.107	0.144	0.152	0.114	0.106	0.101	0.101	0.117	0.120	0.238	0.229	0.121	0.120	0.112	0.115
	Mean F0	7448.5	6527.5	8138.7	8121.5	7301.1	6584.5	7841.2	7382.4	7984.1	7480.4	7942.5	6553.5	8348.6	7999.4	7031.2	6822.1
	F0 start	8067.1	7218.1	8895.4	8867.7	7946.7	7185.6	8477.5	8123.7	8663.7	8205.0	8739.1	7440.5	9047.2	8789.7	7628.1	7398.0
	F0 end	6819.3	5821.7	7067.7	7203.0	6584.6	5843.2	7138.2	6575.1	7314.0	6736.3	7302.9	5894.2	7586.1	7122.9	6417.0	6225.8
	Max F0	8067.1	7219.0	8903.1	8881.2	7959.9	7200.0	8486.0	8124.8	8664.2	8205.0	8751.4	7446.5	9048.2	8790.1	7628.1	7399.3
	% Time Max F0	27.8	28.0	22.8	20.3	25.7	26.2	27.7	29.0	24.0	24.3	18.3	15.5	24.4	24.1	26.3	24.8
	Min F0	6819.3	5821.7	7067.7	7202.4	6580.2	5836.8	7138.2	6574.5	7314.0	6736.2	7297.4	5890.0	7586.1	7122.9	6417.0	6225.8
	F0 abs slope	34734.7	37231.5	33278.0	28902.9	30543.6	28568.0	43676.9	43796.5	27748.5	29509.3	14781.1	13924.2	33416.2	36044.3	31096.8	25747.0
	F0 Var	3816.9	3777.7	6666.6	6844.7	4866.8	5948.4	3726.6	5064.1	5485.9	6172.5	4451.8	6036.7	4856.5	5109.4	3251.4	4042.3
	Q25%	7554.2	6809.7	7887.3	7951.6	7525.0	7731.8	7539.9	7239.5	7636.1	7397.6	7831.4	6836.6	7630.9	7783.1	6872.5	6817.1
	Q50%	9057.1	9160.1	9577.3	9498.3	9773.9	10402.1	9284.7	9100.3	8600.0	8912.2	8919.7	9415.2	9014.5	9292.2	8092.1	8450.8
	Q75%	12558.7	14194.1	13684.2	13964.8	13884.2	13675.0	13272.2	12366.2	11502.0	13466.7	12746.3	13439.9	11630.8	13982.6	11227.0	11862.0
	Fpeak	170.6	175.2	186.6	177.4	164.6	160.8	145.8	187.6	159.3	177.1	197.7	199.9	164.1	187.8	175.5	174.6
	% Time of max intensity	41.6	44.3	44.7	41.5	46.0	48.9	44.0	44.4	41.8	43.8	36.9	36.5	48.7	44.3	43.7	45.1
	jitter	0.032	0.028	0.025	0.024	0.025	0.023	0.027	0.024	0.024	0.026	0.015	0.015	0.023	0.020	0.028	0.024

Table F Average value of each call parameter from the ZH animals in experiment 1 from "*Before*" and "*After2*"

		Kapi		Kapo		Tabor		Thilo		Kaliper		Marlene		Vesta		Vito	
		Before	*After2*	*Before*	*After2*	*Before*	*After2*	*Before*	*After2*	*Before*	*After2*	*Before*	*After2*	*Before*	*After2*	*Before*	*After2*
Trill calls	sound duration	0.472	0.477	0.446	0.387	0.583	0.570	0.498	0.468	0.517	0.501	0.645	0.596	0.415	0.344	0.776	0.360
	Mean F0	6733.6	6149.8	6036.9	5444.6	6376.5	5864.6	6631.9	6542.2	6826.3	6439.8	6561.4	6161.5	5979.2	6013.9	6923.8	6101.2
	F0 start	6198.9	5670.1	5610.8	5196.0	5355.1	4809.7	6019.5	5906.2	6536.9	6177.0	5850.3	5502.3	5632.0	5904.1	5168.3	5736.5
	F0 end	7025.2	6270.3	6086.5	5384.6	6961.5	6417.5	7057.1	6941.5	6877.7	6456.3	6982.8	6472.6	6249.9	6145.6	7712.5	6192.5
	Max F0	7120.7	6437.5	6255.0	5587.1	7101.7	6459.4	7122.8	6978.1	7077.3	6630.1	7037.2	6566.1	6303.6	6221.9	7839.0	6284.5
	% Time Max F0	77.1	67.1	66.7	57.2	73.9	78.6	81.4	79.7	67.4	54.5	77.7	78.2	68.2	64.0	83.0	68.9
	Min F0	6180.1	5630.2	5592.0	5148.9	5292.8	4809.7	6015.0	5904.8	6493.0	6112.3	5842.4	5493.7	5597.5	5847.3	5168.3	5732.3
	F0 abs slope	2958.0	2651.3	2385.0	2190.1	3957.0	3550.2	3105.2	3150.6	2251.0	1904.0	2470.2	2387.1	2165.7	1943.4	4308.5	2322.7
	F0 Var	1883.1	1585.7	1405.3	1187.6	2821.7	2669.2	2126.8	2081.4	1417.1	1191.4	1656.0	1643.9	1242.9	895.0	3348.4	1365.1
	Q25%	8017.7	7940.5	6446.2	6564.9	6876.4	6642.8	6488.2	6471.7	7093.1	6454.5	6702.5	7159.8	6290.2	8032.8	7035.5	6885.2
	Q50%	10822.4	11494.8	9977.1	9669.0	9265.7	9606.7	7861.4	8229.4	8756.7	8679.4	9293.8	9987.3	9563.2	11160.6	8137.6	9966.6
	Q75%	15473.7	14959.2	12508.1	12075.6	12657.5	12252.4	11361.9	11779.1	12689.6	11935.7	12841.3	12271.3	12978.0	14502.4	11919.1	13274.6
	Fpeak	200.6	219.5	214.2	174.8	219.5	205.0	174.0	266.7	229.7	200.1	203.1	224.4	203.2	201.7	338.2	180.6
	% Time of max intensity	22.8	24.4	28.9	30.9	35.2	25.7	26.1	22.2	29.7	29.3	33.5	33.1	28.0	27.5	65.9	24.9
	jitter	0.019	0.022	0.023	0.028	0.019	0.025	0.023	0.019	0.021	0.024	0.024	0.022	0.023	0.024	0.015	0.024
	Fm Rate	2.2	2.4	2.0	2.3	1.5	1.8	2.1	1.7	2.9	2.5	1.8	1.6	2.5	2.8	0.8	2.2
	FM extend	1355.9	1113.7	1117.8	728.5	2822.0	2196.4	1432.3	1621.2	744.3	613.8	1468.3	1579.8	949.6	444.9	4724.1	1030.8
Phee Calls	sound duration	1.089	1.040	0.962	1.172	0.787	1.044	1.231	0.949	1.305	1.374	1.097	1.469	1.963	1.442	1.137	1.546
	Mean F0	7244.2	6809.9	7747.3	7399.6	6979.1	6789.1	7507.5	7506.5	7856.1	6950.7	7690.0	7767.3	7095.3	7409.1	7957.3	8053.2
	F0 start	6518.2	6138.4	6833.5	6381.8	6238.7	5952.1	6623.9	6491.3	7130.3	6368.5	6855.1	7179.0	6511.8	6897.1	7310.6	7055.6
	F0 end	7627.5	7504.7	8119.8	7789.5	6899.9	6885.4	7592.4	8086.8	8168.0	7240.1	8033.1	7906.7	6719.9	7798.6	8165.3	8046.6
	Max F0	7858.1	7590.6	8393.8	8053.4	7450.3	7241.8	8116.8	8231.6	8438.6	7342.0	8272.5	8240.5	7430.9	8091.5	8479.3	8591.8
	% Time Max F0	84.0	85.0	79.4	81.4	71.6	80.2	79.4	83.7	86.6	87.2	77.2	76.8	86.6	76.1	80.5	87.4

	Min F0	6478.8	6101.8	6754.1	6381.1	6190.6	5931.6	6545.7	6491.3	7127.5	6368.5	6791.7	7075.8	6359.1	6608.4	7115.0	6984.6
	F0 abs slope	2146.6	2132.1	2892.4	2327.1	3022.1	2180.8	2200.6	2529.9	1454.3	993.7	2293.4	1299.3	982.2	1683.2	1827.9	1751.9
	F0 Var	1691.5	1606.4	2209.9	1769.0	2202.5	1601.2	1734.4	2003.3	1169.3	792.0	1844.2	1083.1	687.2	1375.0	1483.2	1314.2
	Q25%	7195.4	6710.8	7700.6	7339.8	6976.3	7003.5	7314.8	7265.5	7690.7	6921.2	7479.8	7731.5	7126.9	7301.1	7729.7	7937.6
	Q50%	8151.8	9044.4	8596.7	8157.2	7557.0	7904.7	7713.7	7854.3	7995.7	8650.4	7856.9	8267.6	7333.5	7700.8	8192.1	8258.5
	Q75%	13085.6	13160.3	12133.5	12002.0	10402.2	12576.1	9525.2	10367.2	9097.2	12579.2	8482.6	12761.4	10371.8	11399.9	10460.8	9501.8
	Fpeak	234.9	306.3	233.9	256.5	212.0	258.5	255.2	242.3	257.1	215.0	229.7	317.4	276.7	268.4	253.4	235.7
	% Time of max intensity	46.8	44.0	48.5	48.9	48.0	58.6	39.5	39.8	46.4	45.7	47.6	46.9	52.1	48.4	32.3	49.5
	jitter	0.007	0.005	0.008	0.006	0.006	0.006	0.006	0.009	0.006	0.005	0.006	0.005	0.005	0.008	0.012	0.007
	sound duration	0.099	0.106	0.144	0.150	0.114	0.101	0.101	0.101	0.117	0.136	0.238	0.195	0.121	0.114	0.112	0.107
	Mean F0	7448.5	7100.7	8138.7	8041.1	7301.1	6748.1	7841.2	7743.0	7984.1	8557.2	7942.5	7407.6	8348.6	7826.1	7031.2	7009.6
	F0 start	8067.1	7708.7	8895.4	8907.8	7946.7	7423.4	8477.5	8548.4	8663.7	9430.6	8739.1	8019.8	9047.2	8436.2	7628.1	7601.2
	F0 end	6819.3	6442.6	7067.7	7001.9	6584.6	5895.4	7138.2	6860.2	7314.0	7547.3	7302.9	6930.1	7586.1	7135.7	6417.0	6365.3
	Max F0	8067.1	7708.7	8903.1	8916.6	7959.9	7436.1	8486.0	8549.3	8664.2	9434.1	8751.4	8031.1	9048.2	8447.0	7628.1	7622.7
	% Time Max F0	27.8	27.7	22.8	25.4	25.7	23.7	27.7	28.7	24.0	24.8	18.3	19.4	24.4	24.5	26.3	25.2
	Min F0	6819.3	6442.6	7067.7	7001.9	6580.2	5895.4	7138.2	6859.6	7314.0	7547.3	7297.4	6929.1	7586.1	7135.7	6417.0	6345.6
Food Call	F0 abs slope	34734.7	37533.0	33278.0	37110.2	30543.6	30958.6	43676.9	47140.1	27748.5	34204.0	14781.1	12508.3	33416.2	27700.1	31096.8	27553.7
	F0 Var	3816.9	3158.2	6666.6	4575.7	4866.8	8319.9	3726.6	4628.1	5485.9	6633.9	4451.8	4783.3	4856.5	5133.3	3251.4	5280.4
	Q25%	7554.2	7212.9	7887.3	8186.2	7525.0	7847.3	7539.9	7524.2	7636.1	8376.6	7831.4	7228.6	7630.9	7211.7	6872.5	6959.1
	Q50%	9057.1	9619.1	9577.3	10062.3	9773.9	10495.8	9284.7	9022.0	8600.0	9601.0	8919.7	7992.2	9014.5	8607.5	8092.1	8499.8
	Q75%	12558.7	13933.2	13684.2	15714.6	13884.2	14320.3	13272.2	12336.6	11502.0	12165.4	12746.3	11253.0	11630.8	11347.2	11227.0	12851.8
	Fpeak	170.6	165.3	186.6	172.8	164.6	165.0	145.8	176.5	159.3	160.0	197.7	186.3	164.1	153.1	175.5	191.7
	% Time of max intensity	41.6	45.8	44.7	40.2	46.0	48.5	44.0	44.2	41.8	38.7	36.9	39.2	48.7	51.8	43.7	47.5
	jitter	0.032	0.027	0.025	0.022	0.025	0.020	0.027	0.023	0.024	0.025	0.015	0.017	0.023	0.035	0.028	0.022

Table G Average value of each call parameter from the MA animals in experiment 1 from “*Before*” and “*NewPhys*”.

		Conan		Craken		Washington		Wisconsin	
		Before	*NewPhys*	*Before*	*NewPhys*	*Before*	*NewPhys*	*Before*	*NewPhys*
Trill calls	sound duration	0.274	0.334	0.398	0.647	0.272	0.419	0.214	0.364
	Mean F0	7291.6	8362.9	7684.3	8556.6	7321.6	7063.3	7846.9	7414.4
	F0 start	7337.4	8259.7	7784.6	7883.0	7448.7	6921.6	7905.5	7203.8
	F0 end	7090.6	8427.4	7499.4	8554.0	7218.5	7075.5	7708.4	7430.1
	Max F0	7433.1	8484.5	7944.6	8919.5	7542.4	7176.6	8001.1	7530.7
	% Time Max F0	25.5	70.1	30.5	72.2	25.6	54.0	33.1	52.2
	Min F0	7090.6	8208.9	7381.6	7738.7	7142.6	6894.5	7668.5	7180.8
	F0 abs slope	2511.1	1642.8	2806.3	3257.0	2194.3	1780.9	2585.2	2283.8
	F0 Var	1441.6	945.6	1640.0	2104.5	1568.5	849.2	1664.6	1111.6
	Q25%	7339.3	8279.3	7664.0	8116.9	7296.6	6932.6	7770.0	7230.1
	Q50%	7785.3	9206.0	8149.4	9190.3	7681.2	7663.7	8244.9	7938.1
	Q75%	13205.8	18796.1	12568.3	16116.0	9051.9	13086.0	9795.7	13019.9
	Fpeak	213.0	227.9	206.5	297.0	193.3	202.9	169.9	198.5
	% Time of max intensity	40.1	24.3	28.1	45.8	25.7	22.5	33.1	23.8
	jitter	0.030	0.040	0.034	0.043	0.025	0.043	0.026	0.043
	Fm Rate	2.6	3.7	3.0	3.4	3.8	3.4	3.5	2.7
	FM extend	580.5	406.1	652.2	1094.8	470.8	287.9	638.4	519.7
Phee calls	sound duration	0.422	0.703	0.447	0.987	0.539	0.800	0.532	0.839
	Mean F0	7697.6	8111.2	7680.6	8313.3	7161.8	7417.3	7528.9	7692.3
	F0 start	7557.4	7571.8	7502.7	7554.5	6936.8	6886.9	7309.4	7197.1
	F0 end	7667.3	8338.8	7635.9	8563.0	7138.1	7704.8	7590.5	7996.0
	Max F0	7923.2	8412.0	7973.5	8749.5	7415.9	7803.0	7775.5	8082.4
	% Time Max F0	54.7	75.0	37.5	72.5	60.9	79.9	66.9	79.6
	Min F0	7358.9	7548.5	7248.5	7514.8	6813.9	6866.3	7231.7	7181.2
	F0 abs slope	2319.4	1680.3	2720.1	1966.7	2073.9	2315.3	1789.8	2098.0
	F0 Var	1726.2	1337.3	1836.2	1489.8	1707.9	1408.8	1476.2	1447.5

	Q25%	7699.6	7932.2	7560.5	8001.7	7044.7	7174.2	7396.2	7503.3
	Q50%	8080.9	8290.3	8373.5	8585.8	7263.1	7455.5	7633.5	7849.5
	Q75%	12431.5	10284.2	12309.1	11570.2	8183.9	8530.1	9562.5	10562.4
	Fpeak	205.5	219.7	225.3	242.3	210.1	239.1	219.1	227.3
	% Time of max intensity	35.5	42.9	37.5	33.4	41.6	27.6	44.5	36.9
	jitter	0.029	0.016	0.028	0.025	0.011	0.019	0.019	0.021
Food calls	sound duration	0.064	0.090	0.078	0.093			0.084	0.101
	Mean F0	7892.3	8259.3	7543.4	7263.6			7623.8	7994.9
	F0 start	8239.8	8574.1	7905.2	7639.9			8037.4	8388.4
	F0 end	7471.5	7914.4	7122.8	6860.8			7211.5	7592.3
	Max F0	8281.6	8576.9	7975.7	7648.4			8050.8	8388.9
	% Time Max F0	21.7	27.1	23.8	24.6			20.0	29.6
	Min F0	7435.6	7914.4	7118.1	6860.8			7206.4	7592.3
	F0 abs slope	25835.3	23996.9	19278.0	31121.8			17044.9	27056.9
	F0 Var	8636.1	1595.3	8047.0	1183.5			7333.6	1263.7
	Q25%	7406.3	7356.7	7332.2	7349.6			7294.0	7290.3
	Q50%	8221.8	8209.2	7953.1	8882.0			8100.4	8294.2
	Q75%	9762.2	9537.9	10549.3	14215.8			11086.8	11320.9
	Fpeak	161.9	165.9	146.0	163.0			153.0	163.9
	% Time of max intensity	50.6	47.6	47.0	43.6			48.0	44.7
	jitter	0.030	0.049	0.035	0.053			0.030	0.041

Table H Average value of each call parameter for each MA individual in the situation "*Before*" and "*NewSoc*" as well as of the ZH animals (Baseline) involved in experiment 2.

		Conan		Craken		Washington		Wisconsin		Lilly	Gatto	Mibba	Nautilus
		Before	*NewSoc*	*Before*	*NewSoc*	*Before*	*NewSoc*	*Before*	*NewSoc*	*Baseline*	*Baseline*	*Baseline*	*Baseline*
Trill calls	sound duration	0.274	0.634	0.398	0.738	0.272	0.516	0.214	0.502	0.570	0.369	0.702	0.808
	Mean F0	7291.6	7254.5	7684.3	7273.1	7321.6	7010.8	7846.9	7355.6	6609.6	6684.0	6652.1	7042.3
	F0 start	7337.4	6704.6	7784.6	6399.2	7448.7	6607.6	7905.5	6901.5	6191.9	6439.6	5956.3	6320.6
	F0 end	7090.6	7296.5	7499.4	7567.4	7218.5	7120.6	7708.4	7439.2	6688.2	6769.2	6812.9	7393.1
	Max F0	7433.1	7564.9	7944.6	7697.1	7542.4	7217.1	8001.1	7553.6	6957.9	6836.4	7090.9	7555.6
	Perc Time Max F0	25.5	63.0	30.5	74.9	25.6	69.2	33.1	65.3	62.2	64.3	65.9	74.8
	Min F0	7090.6	6647.6	7381.6	6393.9	7142.6	6603.7	7668.5	6886.9	6086.9	6432.9	5826.7	6284.5
	F0 abs slope	2511.1	2639.1	2806.3	2394.3	2194.3	1989.7	2585.2	2342.2	2831.2	2052.6	2629.1	2471.9
	F0 Var	1441.6	1625.9	1640.0	1776.7	1568.5	1212.4	1664.6	1395.5	1688.8	1114.4	1961.8	1666.5
	Q25perc	7339.3	7077.8	7664.0	6977.1	7296.6	6821.8	7770.0	7093.7	8148.4	6731.1	6787.9	7123.6
	Q50perc	7785.3	7797.6	8149.4	7583.1	7681.2	7455.9	8244.9	7579.8	9878.8	8633.1	9234.4	8072.6
	Q75perc	13205.8	10030.5	12568.3	9888.1	9051.9	10284.9	9795.7	9482.6	12738.7	13279.8	13103.2	12240.7
	Fpeak	213.0	201.6	206.5	191.4	193.3	186.1	169.9	186.9	235.9	210.3	236.1	220.9
	Perc Time of max intensity	40.1	26.3	28.1	30.0	25.7	23.1	33.1	22.2	33.3	26.9	24.7	54.4
	jitter	0.030	0.030	0.034	0.027	0.025	0.040	0.026	0.037	0.026	0.023	0.016	0.022
	Fm Rate	2.6	2.8	3.0	2.6	3.8	2.9	3.5	2.7	2.8	2.9	1.7	1.9
	FM extend	580.5	1026.5	652.2	1119.0	470.8	610.3	638.4	721.5	1244.1	525.3	1843.6	1855.9
Phee calls	sound duration	0.422	0.725	0.447	1.378	0.539	1.502	0.532	1.182	0.863	0.757	0.955	0.927
	Mean F0	7697.6	8006.5	7680.6	7660.0	7161.8	7464.9	7528.9	7344.7	7141.2	7296.1	7692.7	7512.6
	F0 start	7557.4	7422.0	7502.7	6864.4	6936.8	6585.0	7309.4	6627.7	6348.5	6609.7	6699.9	6625.7
	F0 end	7667.3	8165.3	7635.9	7777.7	7138.1	7859.9	7590.5	7619.1	7614.9	7226.1	8047.1	7793.5
	Max F0	7923.2	8300.8	7973.5	8065.5	7415.9	8024.8	7775.5	7872.2	7715.2	7656.6	8358.7	8224.8
	percTime Max F0	54.7	74.8	37.5	74.9	60.9	85.1	66.9	78.9	81.5	64.5	82.0	79.8
	Min F0	7358.9	7391.8	7248.5	6817.4	6813.9	6571.9	7231.7	6604.9	6316.7	6572.9	6673.8	6576.4

	F0 abs slope	2319.4	1847.3	2720.1	1612.6	2073.9	1422.6	1789.8	1943.4	2240.0	2580.2	2503.8	3049.4
	F0 Var	1726.2	1432.9	1836.2	1284.5	1707.9	1161.3	1476.2	1543.2	1807.5	2018.8	2012.2	2204.6
	Q25perc	7699.6	7826.0	7560.5	7471.7	7044.7	7218.2	7396.2	7094.9	7187.4	7176.9	7582.9	7363.4
	Q50perc	8080.9	8179.0	8373.5	7785.7	7263.1	7465.5	7633.5	7386.1	8079.7	7436.9	8285.8	7725.3
	Q75perc	12431.5	9817.2	12309.1	9009.0	8183.9	7906.2	9562.5	8298.8	12734.9	7912.3	11777.0	9214.9
	Fpeak	205.5	188.1	225.3	214.6	210.1	218.4	219.1	215.4	224.7	218.5	231.1	253.2
	percTime of max intensity	35.5	43.9	37.5	49.5	41.6	40.7	44.5	40.2	58.9	46.8	53.4	51.5
	jitter	0.029	0.018	0.028	0.014	0.011	0.011	0.019	0.012	0.004	0.005	0.007	0.007
Food Calls	sound duration	0.064	0.115	0.078	0.132	0.084	0.141	0.084	0.143	0.156	0.105	0.122	0.135
	Mean F0	7892.3	8484.7	7543.4	7783.6	7830.2	9300.1	7623.8	7860.0	7947.8	7549.6	7955.0	6785.4
	F0 start	8239.8	8894.7	7905.2	8221.4	8157.1	9973.1	8037.4	8375.3	8791.3	7989.7	8519.9	7603.9
	F0 end	7471.5	7814.5	7122.8	7144.2	7502.7	8355.4	7211.5	7274.8	6770.2	7045.9	7227.1	5798.9
	Max F0	8281.6	8969.2	7975.7	8287.1	8164.7	9990.9	8050.8	8396.3	8853.4	8002.4	8542.4	7616.5
	percTime Max F0	21.7	28.3	23.8	25.8	19.8	23.8	20.0	22.0	21.3	24.4	21.4	23.3
	Min F0	7435.6	7798.4	7118.1	7133.4	7499.6	8355.4	7206.4	7264.7	6764.4	7041.9	7222.3	5798.9
	F0 abs slope	25835.3	26740.0	19278.0	22176.7	11980.1	28653.7	17044.9	20804.3	30917.6	26234.2	25412.5	36039.0
	F0 Var	8636.1	4804.7	8047.0	4422.5	6319.8	6115.8	7333.6	4172.0	9012.8	3988.3	6376.6	5804.9
	Q25perc	7406.3	8252.5	7332.2	7531.6	7485.0	8544.7	7294.0	7530.3	8161.5	7445.0	7803.0	6977.1
	Q50perc	8221.8	9237.8	7953.1	8430.4	8094.5	9502.0	8100.4	8677.6	10155.3	8569.0	9449.9	9113.9
	Q75perc	9762.2	11469.7	10549.3	10405.1	9573.3	11227.9	11086.8	11027.2	14319.6	11181.6	13796.3	12628.6
	Fpeak	161.9	152.2	146.0	147.4	156.9	158.4	153.0	154.2	175.7	171.2	164.8	179.1
	percTime of max intensity	50.6	46.9	47.0	43.2	47.5	47.8	48.0	40.3	40.3	43.1	45.2	44.2
	jitter	0.030	0.026	0.035	0.026	0.020	0.023	0.030	0.027	0.019	0.024	0.023	0.034
